# Applying Subcritical Water Extraction to Obtain Bioactive Compounds and Cellulose Fibers from Brewer Spent Grains

**DOI:** 10.3390/molecules29204897

**Published:** 2024-10-16

**Authors:** Paula Andrea Gomez-Contreras, Catalina Obando, Pedro Augusto Vieira de Freitas, Laia Martin-Perez, Amparo Chiralt, Chelo Gonzalez-Martinez

**Affiliations:** Institute of Food Engineering FoodUPV, Universitat Politècnica de València, Camino de Vera s/n, 46022 Valencia, Spain; pagomco1@doctor.upv.es (P.A.G.-C.); cobagar@upvnet.upv.es (C.O.); pedvidef@doctor.upv.es (P.A.V.d.F.); lmarper1@upv.es (L.M.-P.); dchiralt@tal.upv.es (A.C.)

**Keywords:** phenolic compounds, cellulose fibres, antioxidant, antimicrobial, hydrogen peroxide bleaching

## Abstract

Of the three types of waste generated in beer processing, brewer’s spent grain (BSG) is the most abundant and has a high potential for valorization. In this work, defatted BSG (DB) was subjected to an extraction process with subcritical water at different temperatures to obtain extracts rich in phenols and the cellulosic fractions, which were also purified by using hydrogen peroxide (H_2_O_2_). The results showed that the dry extracts obtained at 170 °C were richer in phenolics (24 mg Gallic Acid Equivalent (GAE) g^−1^ DB), but with lower antioxidant capacity (71 mg DB·mg^−1^ 2,2-diphenyl-1-pikryl-hydrazyl). This extract also showed the highest antibacterial potential against *L. innocua* (80 mg·mL^−1^) and *E. coli* (140 mg·mL^−1^) than those obtained at lower temperatures. The purification of cellulose from the treated residues, using hydrogen peroxide, revealed that DB is a limited source of cellulose material since the bleached fractions showed low yields (20–25%) and low cellulose purity (42–71%), even after four bleaching cycles (1 h) at pH 12 and 8% H_2_O_2_. Despite this, the subcritical water extraction method highlights the potential of a simple process as a technological option to convert underutilized side streams like beer bagasse into added-value, potential ingredients for innovative food and pharmaceutical applications.

## 1. Introduction

Global beer consumption has experienced a notable increase over the last 50 years, reaching 150 billion liters per year, surpassing wine consumption by seven [1]. It is estimated that by 2025, it will be worth $502.9 billion and have an annual growth rate of 19.9% in response to growing demand and the wide variety of new styles and flavors [2]. During the beer brewing process, three types of waste are generated: brewer´s spent grain (BSG or beer bagasse), spent hops, and spent brewer’s yeast. It is estimated that between 14 and 20 kg of bagasse, 0.2 and 0.4 kg of hops, and 1.5 and 3 kg of residual yeast are generated for every 100 L of beer produced [1]. Consequently, the beer bagasse constitutes approximately 85% of the total byproducts generated [3]. This residue presents outstanding nutritional properties that make it a valuable source of high-value compounds and includes hulls of barley malt grains, parts of the pericarp, and layers of the barley seed coat. It contains fibers (soluble and insoluble), proteins, lipids, and phenolic compounds, which can be free or bound to dietary fiber [4]. Among phenolic acids, ferulic, p-coumaric, and caffeic predominate in a bound state [5]. The chemical composition of BSG depends on the type of barley, the time of harvest, and the malting and mashing conditions, being rich in cellulose (12–25% in dry basis (db)), hemicellulose (20–25%), lignin (10–28%), starch (2–7%), proteins (20%), lipids (6–10%), and minerals (2–5%) together with polyphenols (0.7–2%) [5,6]. Therefore, the extraction of different fractions from beer bagasse could be of great interest to obtain different valued biomolecules to be used for the pharmaceutical, packaging, or food industry.

The extraction of different compounds from BSG has been studied by a few researchers for different purposes, which were mainly focused on the recovery of proteins and polyphenols. Thus, some authors [7] reviewed the effect of extraction techniques and conditions on the composition, physicochemical, and techno-functional properties of the obtained BSG protein extracts. Other authors [8] focused on the current extraction methods used to obtain phenolic compounds from BSG, ranging from more traditional (the conventional solid-liquid extractions employing organic solvents, alkaline, and enzymatic reactions) to advanced techniques such as pressurized fluid, supercritical, microwave-assisted, and ultrasound-assisted extractions. On the other hand, a great yield of phenols (28.9 g gallic acid/100 g BSG) and a high percentage of the lignin (54.4%) and protein (79%) present in the BSG were reported by [9], using different deep eutectic solvents and temperatures.

Among the various new green extraction and separation technologies, ultrasound-assisted, microwave-assisted, enzyme-assisted, and subcritical and supercritical fluid extraction have been recently used, surpassing conventional methods such as maceration, infusion, and Soxhlet extraction [10,11]. In subcritical water extraction (SWE), where water is liquid at the temperature and pressure below its critical point (374.15 and 22.1 MPa) [12], the physicochemical properties of water, such as the relative dielectric constant and polarity, decrease significantly with increasing temperature so that, under subcritical conditions, water can dissolve polar and non-polar compounds [13]. Furthermore, viscosity and surface tension decrease with increasing temperature, resulting in higher extraction efficiency [14,15]. Additionally, water is an economical, non-toxic, non-hazardous, and safe solvent that works as a solvent and catalyst to take advantage of and transform biomass into bioactive products [16,17]. Compared with organic solvents, subcritical water not only has advantages in ecology, economy, and safety, but also its density, viscosity, ion product, and dielectric constant can be adjusted by temperature, thus promoting the extraction of compounds with different polarity with no residue or effluents and high environmental friendliness. In the last few years, this technology has shown an increasing interest in the scientific community (from 300 articles published in 1995–2000 to 5000 articles in the 2021–2023 period), thus showing SWE is a promising technology for extracting target compounds such as proteins, polyphenols, or phenolic acids from different sources.

SWE has been applied in a variety of agro-industrial wastes such as winery, tea, shellfish, and tobacco wastes, carrot leaves, cotton flowers, peels (chestnut, almond, potato, citrus, mandarin, mango, kiwifruit), seeds (papaya), pomace (apple, pomegranate, kiwifruit), among others, to recover different bioactive active compounds such as querquecin, flavonoids, different phenolic acids, and anthocyanins, but also polysaccharides such as pectins, proteins, and peptides [18]. Some authors [19] used SWE as a hydrolytic medium to recover proteins and specific polyphenols from craft BSG for pharmaceutical and cosmetic applications. They found 185 °C to be the best temperature to maximize the extraction of protein and aldehyde phenolic compounds (vanillin, syringic, and protocatechuic aldehydes), while lower temperatures (160 °C) promoted the extraction of hydroxycinnamic acids, such as ferulic acid and p-coumaric.

In addition, the extraction of bioactive compounds and the cellulose recovery from beer bagasse have also been studied. Thus, [20] addressed the extraction of cellulose from BSG by means of alkaline hydrolysis and bleaching reactions and its further conversion into cellulose acetate for packaging applications. The purification of cellulose from a beer industrial residue from 40% to 92% by using different steps of pulping and chlorine bleaching has been studied by [21], which considered bagasse as an interesting source of cellulose. Nevertheless, in the cellulose purification process from lignocellulosic residues, great amounts of chemicals are involved, which are necessary to reduce in order to minimize its environmental impact. Combination of SWE and milder bleaching treatments with oxygen derivatives, such as hydrogen peroxide, could be an interesting alternative to purify cellulose. No previous studies have been found by applying SWE combined with mild bleaching treatments to obtain in cascade phenolic-rich extracts from the SWE step and cellulose fibers from the subsequent bleaching step of the SWE extraction residue.

The objective of the present work is to fractionate the beer bagasse residue into phenolic and cellulosic-rich fractions by applying subcritical water extraction (SWE) at different temperatures (110–170 °C) and subsequent hydrogen peroxide bleaching treatments of the extraction residues. The SWE extracts were analyzed as to their compositional, antioxidant, and antibacterial properties, while the cellulose purification degree was quantified in the insoluble residues submitted to different bleaching steps. Thus, an integral fractionation of added value products from BSG waste was proposed, which will contribute to valorizing the BSG waste in the context of a circular economy.

## 2. Results and Discussion

### 2.1. Yields and Composition of the Different SWE Fractions

**Figure 1:**
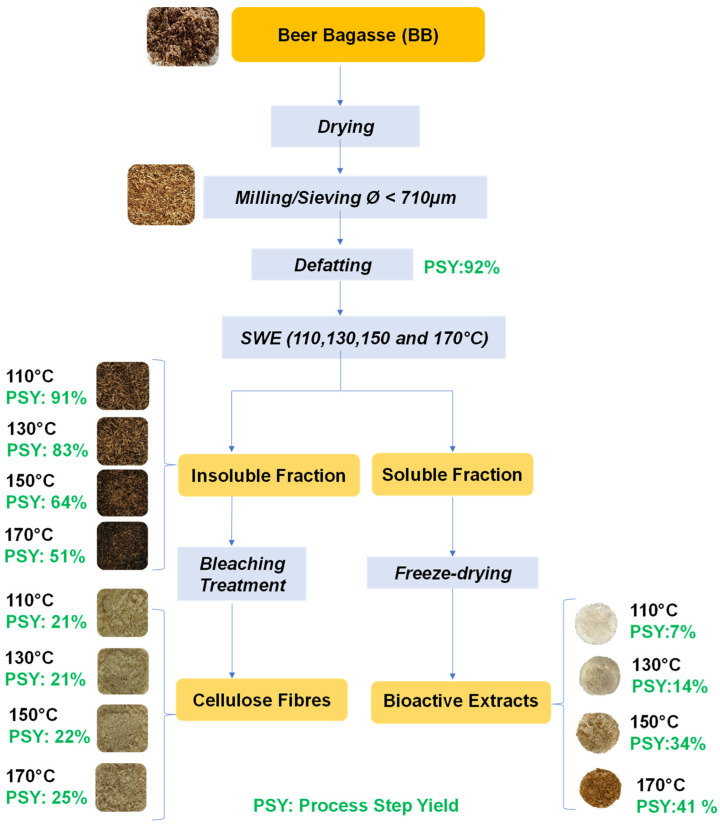
Flow chart of the beer bagasse fractionation and the process step yields (PSY: g outgoing solids). 100 g^−1^ of incoming dried material) for the defatting step and SWE carried out at the different temperatures.

Figure 1 shows the flow chart of the brewer spent grain (BSG) or beer bagasse fractionation throughout the SWE, giving rise to soluble extracts (E) and insoluble residues (R) at each temperature. The latter were submitted to a bleaching step to purify cellulose, while the extracts were freeze-dried to obtain extract powders. Images of the different obtained products, together with the obtained mass yield of each process step, are also shown in Figure 1. Previously to the SWE, a defatting step of the BSG yielded around 8% oil from the dried bagasse, being this value in agreement with that reported in the literature [22,23]. The main beer bagasse lipid compounds have been reported, these being triglycerides (55–67%), free fatty acids (18–30%), such as palmitic, oleic, and linoleic acids, and free steroids (5%), such as sitosterol and campesterol. These lipid compounds have a wide range of nutraceutical, pharmaceutical, and cosmetic applications [23].

The values of mass yields of solid extracts (E-110, E-130, E-150, and E-170) and dried residues (R-110, R-130, R-150, and R-170) of the SWE process performed at different temperatures, shown in Figure 1, indicate that the extraction yield increased (from 7% to 41%) when the extraction temperature rose from 110 °C to 170 °C. This is mainly explained by the changes in the water solvent properties when the temperature increased, which reduces the strength of hydrogen bonds and leads to an important reduction in dielectric constant, this becoming closer to the dielectric constant value of some organic solvents, such as methanol (ε = 33) or ethanol (ε = 25) [24,25]. The sum of both yields (extract and residue) at a given temperature closed the mass balance, thus indicating a low mineralization degree of the organic matter present at the processing conditions used.

**Figure 2:**
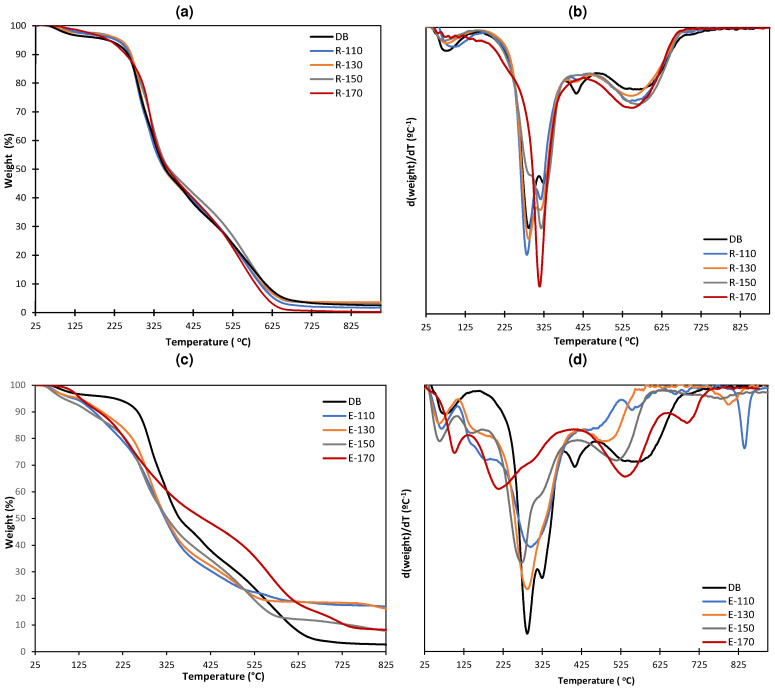
TGA (**a**,**c**) and DTGA (**b**,**d**) of the defatted beer bagasse (DB), the active extracts (E), and the insoluble fractions (R) obtained from SWE at different temperatures.

The thermogravimetric analysis (TGA) curves of the defatted bagasse (DB), extracts, and residues obtained after SWE at different temperatures are shown in Figure 2a, together with the derivate curves (DTGA) (Figure 2b). The DB presented three main degradation steps: the first mainly corresponding to the loss of bound water; a second step associated with the degradation of polysaccharides with different thermostability such as hemicelluloses (150–350 °C), celluloses (275–350 °C), and a part of lignin (160–900 °C); and the third one, related to the degradation of residual lignin and secondary metabolites from the previously thermo-degraded compounds, as previously described by other authors for lignocellulosic biomass [26,27]. The major weight losses took place between 225 and 625 °C (80%), in line with the lignocellulosic nature of this residue, in agreement with the results obtained by other authors [27,28]. Very similar TGA patterns were obtained for every lignocellulosic residue. Nevertheless, it is remarkable that the highest extraction temperatures (150 and 170 °C) gave rise to the samples with the highest peak temperatures (temperature of the maximum degradation rate), which indicates that these were the most enriched in cellulose that shows peak temperature between 330–350 °C [29]. In contrast, the TGA and DTGA curves of the extracts revealed a more complex compositional profile, exhibiting several thermodegradation steps. The extract obtained at the highest temperature showed a higher proportion of compounds that degrade at higher temperatures according to a greater extraction of polymeric components, such as hemicellulose or lignin. A higher final mass residue was also observed in comparison with the untreated sample (defatted bagasse), which can be due to the extract enrichment in minerals or formation of degraded organic matter from the soluble compounds.

Some compositional differences in the extracts and residues obtained at each temperature can be observed in Table 1 and Table 2.

In Table 1, the total (water and ethanol) extractive content, protein, ashes, cellulose, hemicellulose, and acid-insoluble lignin contents of the DB can be observed, together with the values obtained for the different SWE solid residues. The obtained values for raw brewer´s spent grain were within the range previously reported (around 16–22% for cellulose, 24–28% hemicellulose, and 9–27% total lignin) [30,31]. The cellulose content of the DB would be overestimated due to the hemicellulosic glucose contribution, whereas the hemicellulose would be underestimated by the used quantification method. Thus, the greater the hemicellulose content, the higher the quantification error. Likewise, the presence of β-glucan and starch could also contribute to the glucose analyzed. Nevertheless, the content of these components would be negligible after wort production due to their solubilization promoted by enzymatic action from the grains [5].

In the extraction residues, the hemicellulose content was very low at temperatures greater than 150 °C, in accordance with the selective dissolution of hemicellulose under the subcritical water conditions [32,33]. Thus, the hemicellulose started to be removed from the beer bagasse matrix when using temperatures greater than 130 °C, reaching very low values at 170 °C (2%). At these temperatures, the lignin content significantly increased, which confirmed that this fraction of the biomass was not released under SWE, as it has been previously observed by other authors [32]. On the other hand, the increment in the cellulose significantly increased (*p* < 0.05) in the residues treated at the highest temperature (R-170) in comparison with the untreated DB. The insoluble-acid lignin in the solid residues accounted for 87, 101, 99, and 160% of the total lignin in the raw material for 110, 130, 150, and 170 °C, respectively. Thus, the obtained lignin values are surely overestimated, as the outcome of such gravimetric analysis is highly disturbed by the presence of non-lignin acid-insoluble material, e.g., proteins [34]. The corrected lignin (calculated by subtracting the protein content) was not given because, in most cases, negative values were obtained. According to [32], changes in the lignin structure took place during the SWE, such as condensation reactions and structural alterations.

In both extract and residue fractions, the greater mass loss in TGA curves was observed for the temperature range of 200–700 °C, where the lignin is mainly degraded, in line with the formation of secondary metabolites from the previously thermo-degraded compounds. This thermal degradation behavior agreed with that found in the literature for other lignocellulosic residues [26].

Therefore, the application of SWE led to a selective fractionation of DB, giving rise to aqueous extracts richer in different compounds of lower molecular weight (sugars, phenolic compounds, and minerals) and polysaccharide and lignin-rich insoluble residues.

The ash content of the DB, extracts, and residues (shown in Table 1 and Table 2) showed that minerals were mainly present in the insoluble residues, whereas small amounts were released to the extracts. The value obtained per DB was in the range of the ash content reported by other authors for beer spent grain (2–5 g/100 g dry DB), being the most abundant constituents phosphorous, magnesium, calcium, and potassium [30].

In Table 1, the protein content of DB is also shown (around 22%), being this value in the range of previously reported values for beer bagasse, considering a fat-free basis [1,19]. The partition of the protein content during the SWE gave rise to greater content in the insoluble residues, thus suggesting a low solubility of the bagasse proteins under the used water subcritical conditions, especially at the lowest SWE temperatures. At 110 °C, 95% of the total protein remained in the insoluble residue, with this percentage decreasing to around 53% at 170 °C. These proteins are extracted and/or hydrolyzed during the thermal treatment, leading to peptide chains of different sizes or free amino acids or even amino acid decomposition, especially at high temperatures, producing different carboxylic acids and other nitrogen-containing compounds such as ethanolamine [35,36]. Therefore, SWE treatment of beer bagasse can be considered an efficient extraction method to recover the protein fraction of the BSG generated in the beer industry, with the maximum recovery of solubilized protein in the SWE extracts being 47% at 170 °C. This yield was similar to that obtained by the traditional alkaline extraction process (41%), and lower than the obtained using deep eutectic solvents (79%) [9,37].

### 2.2. Functional Properties of the SWE Extracts: Antioxidant and Antibacterial Properties

Total phenolic content (TPC) and antioxidant activity of the aqueous extracts obtained from the SWE process at different temperatures are displayed in Table 2. The antioxidant activity of the different extracts was determined through the total phenolic content by the Folin–Ciocalteu method and the Efficient Concentration parameter (EC_50_) with DPPH radical, which quantifies the amount of extract needed to reduce the initial concentration of the radical up to 50%. The TPC determined in the solid extracts (TPC_1_) was also expressed per mass unit of defatted bagasse (TPC_2_). The TPC values increased from 16 to 59 mg GAE/g dried extract as the extraction temperature rose. Similarly, other authors [38] observed that BSG aqueous extract obtained at 160 °C showed the highest TPC values than that obtained at 100 and 140 °C. The found TPC values in defatted bagasse (7.7 mg GAE/g DB) are in the range of those reported by other authors (0.89–15 mg GAE/g sample), which is highly affected by the solvent and extraction method used [39]. As reported by several authors [5,19,40,41], the main phenolic compounds identified in the BSG extracts were hydroxycinnamic acids (specifically, ferulic acid and p-coumaric acid), sinapic, caffeic, and syringic acids to a lesser degree. The TPC values of the extracts expressed per mass unit of DB (TPC_2_ in Table 2) increased with the extraction temperatures from 1.2 to 24 mg GAE/g DB, the extracts obtained at 170 °C exceeding the values determined in the raw material. These TPC values were greater than those reported by other authors [9,39], using different organic solvents and deep eutectic solvents (0.04–1.1 and 2.89 mg GAE/g BSG, respectively), which indicates the high efficiency of SWE to extract phenols, mainly at temperatures between 150–170 °C. This high efficiency can be attributed to the promotion of hydrolysis of lignin/phenolics-carbohydrate complexes, fostering the decomposition of these structures and releasing free phenolics. Likewise, the neoformation of antioxidant compounds under severe SWE conditions has also been described [42,43]. This neo-formed antioxidant compound could also be quantified as phenols by the unspecific Folin-Ciocalteu reagent. These compounds are formed through Maillard and/or caramelization reactions, producing 5-hydroxymethylfurfural (HMF) and sugar condensation compounds, and exhibit different bioactivities, including antioxidant activity [31]. On the other hand, the thermal degradation of the phenolic compounds at high temperatures could also occur. Specifically, flavonoids in the beer bagasse are highly thermosensitive. Therefore, the extent of the different phenomena that occurred during SWE, depending on the composition of each natural matrix, will determine the final content and nature of phenolics in the extracts. Thus, the marked increment in the TPC observed at 170 °C could be attributed to the large progression of the hydrolytic phenolic release, compared to the potential degradation ratio, as well as to the neo-formation of higher amounts of antioxidant species.

The antioxidant capacity, measured throughout the EC_50_ values, is also shown in Table 2. This value increased when the temperature rose, thus indicating a decrease in the radical scavenging capacity of the extracts. This decrease in the antioxidant capacity when the temperature rose, despite the promotion of higher phenolic content, can be attributed to the different phenolic profile in each extract with different redox potential. The different redox potentials of the antioxidants in the extracts may explain this apparent discrepancy, since some compounds can be reduced by the Folin reactant but not by the DPPH radical. On the other hand, for the colorimetric methods used to determine antioxidant capacity, the chemical prediction is difficult in many cases due to the complex kinetics and stoichiometries. In fact, different studies [39,44] no significant correlation between the TPC determined by a redox method, such as Folin Ciocalteu, and the antioxidant capacity of the extracts against different radicals, such as DPPH.

The antimicrobial potential of the DB extracts was also studied against the Gram-negative *E. coli* bacteria and the Gram-positive *L. innocua*, which are well-known pathogenic microorganisms responsible for food poisoning. The minimal inhibitory concentrations (MIC values) of the extracts with both bacteria were determined and shown in Table 2. The antibacterial effectiveness increased when the extraction temperature rose, making the gram-positive bacteria (Listeria) more sensitive to the extracts. The E-170 MIC value for *E. coli* was similar to that found for SWE extract of almond peel (90 mg/mL) obtained at 160 °C [26]. The antimicrobial efficiency of the polyphenols from the brewery waste stream against *S. aureus*, *L. monocytogenes*, *Salmonella* spp., and *E. coli* bacteria has also been reported by [45], being ferulic and caffeic acids and flavonoids the main responsible for the observed antimicrobial activities.

The obtained results indicate that the SWE extracts from beer bagasse are excellent candidates to be used as antioxidants in the development of a broad spectrum of enriched food stuff such as fortified snacks, yoghurts, juices, or beverages, among others, with the aim of increasing their antioxidant potential or in the pharmaceutical sector. Furthermore, they can be employed as antilisteria compounds in food preservation, especially in the production of meat products, such as sausages, pate sausages, hot dogs, and ready-to-eat foods. The incorporation of these extracts into food packaging materials could also be interesting to protect the food products against oxidation and/or listeria growth.

### 2.3. Bleaching of the Extraction Residues

**Figure 3:**
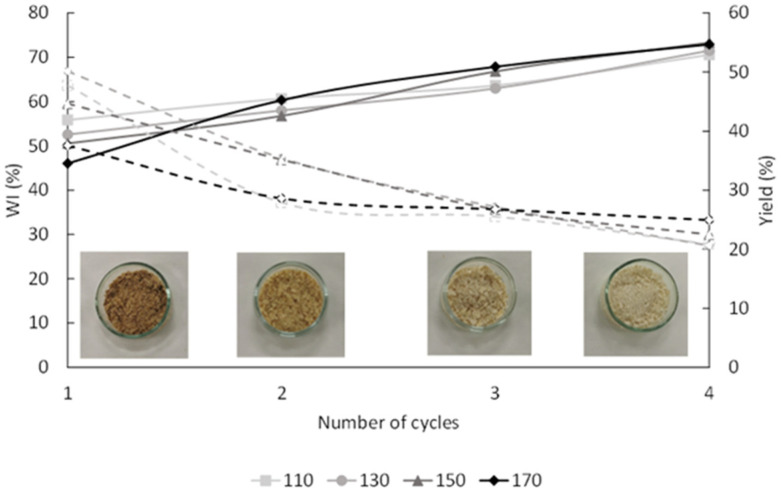
Visual appearance, white index (WI) (full lines), and Yield (%) (dashed lines) of the insoluble residues submitted to different bleaching cycles.

The extraction residues (R-110, R-130, R-150, and R-170) were bleached to recover the cellulose fraction (BR-110, BR-130, BR-150, and BR-170), as they can be used for different applications in the material development and pharmaceutical sectors. The bleaching treatment was carried out using a greener bleaching agent than the usual chlorine bleaches to minimize the environmental impact of the process. Thus, the insoluble fractions were submitted to four successive 1 h cycles with a 4% H_2_O_2_ solution at pH 12. To evaluate the efficiency of the process, the white index (WI) and the yield of the process were determined in cycle for the different samples (Figure 3). As expected, the application of four successive bleaching cycles significantly decreased yield and increased the white index (WI) values, in accordance with the progressive purification of cellulose in each cycle.

The cellulose purification progress was monitored through the analysis of lignin and sugars by means of the NREL method [46,47]. After removal of the water (which includes soluble sugars) and ethanol extractives in the samples (between 13–34%), the acid-insoluble lining and hydrolyzed sugars were quantified in the different samples. Glucose was the major component, followed by xylose and arabinose. As established in the NREL method, hemicellulose content was considered as total xylose and arabinose, and total glucose was attributed to the cellulose content. The obtained values are given in Table 3.

The hemicellulose content was selectively removed when successive bleaching cycles were applied (*p* < 0.05) in every sample. This hemicellulosic fraction significantly decreased when using 3–4 cycles in BR-110 and BR-130 samples and completely disappeared in BR-150 and BR-170 samples after two and one bleaching cycles, respectively. Nevertheless, no significant increase in the cellulose content of the samples occurred during the successive cycles, except for R-170, in which the cellulose content significantly decreased with successive cycles. This suggests that cellulose is progressively degraded through the bleaching cycles with hydrogen peroxide. In fact, when referring to the cellulose content per mass unit of initial DB (Table S2, Supplementary Materials), a progressive decrease was observed, ranging from 14–17 cellulose/100 g DB in the non-bleached residues to 7–13 g cellulose/g DB in the fourth bleaching cycle of the different samples. Degradation of cellulose by the oxidative action of hydrogen peroxide has been reported by other authors [48], through free radical mechanisms forming alpha-hydroxyalkyl radicals and subsequent chain scission. This process is largely affected by the substrate composition and the presence of catalyzers or inhibitors of the reaction. Therefore, the use of hydrogen peroxide as a bleaching agent of BD cellulosic fractions did not yield proper results since an important part of cellulose is degraded during the delignification process.

The maximum cellulose content (about 64%) was obtained in the samples treated at 150 °C after two successive cycles of bleaching. Other authors [20,49] reported 60–95% of cellulose content in bleached BSG, depending on the extraction technique, pretreatment applied, and process condition used, but using more toxic and corrosive agents to carry out alkaline and acid hydrolysis. 

In general, the acid-insoluble lignin content decreased when successive cycles were applied, especially after the fourth bleaching cycle. Nevertheless, as commented on above, it has to be taken into account that these values were affected by the protein content of the samples. As can be observed, the protein is progressively removed by successive bleaching cycles, especially in sample BR-170, where higher protein solubilization occurred in the SWE step.

**Figure 4:**
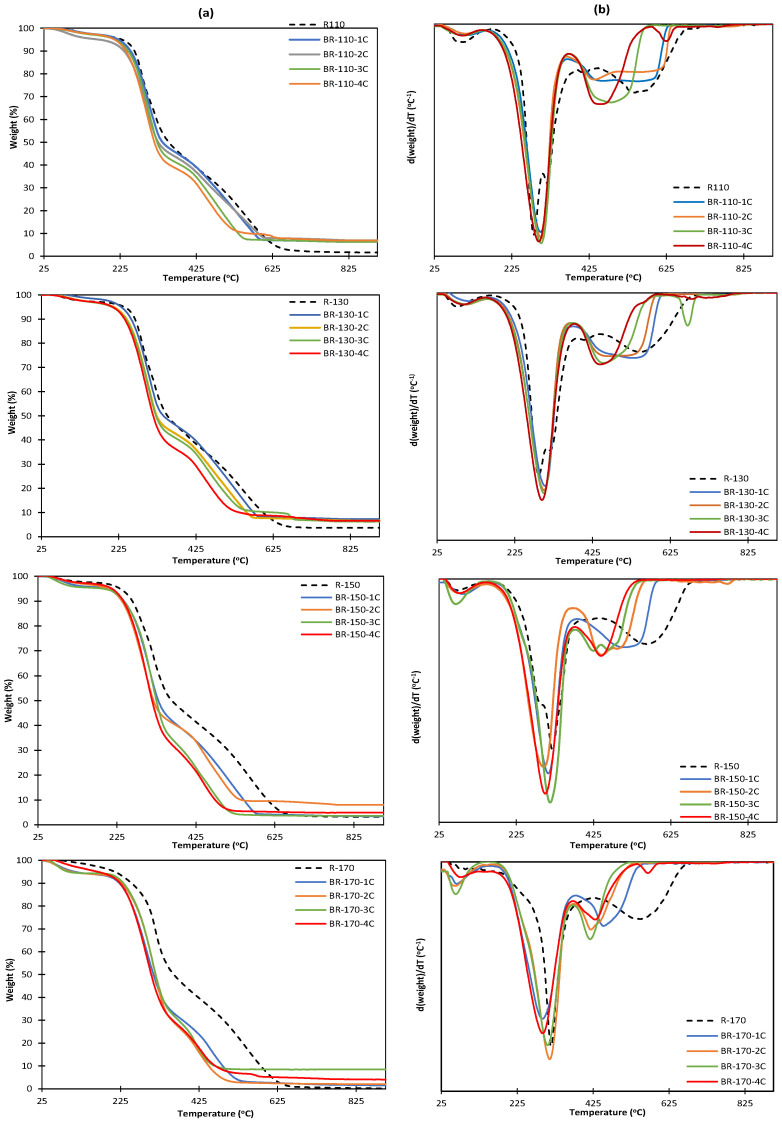
TGA (**a**) and DTGA (**b**) curves of the insoluble residues (R) and the bleached residues (BR) obtained from SWE at 110 °C, 130 °C, 150 °C, and 170 °C submitted to different bleaching cycles.

The TGA and DTGA curves of the insoluble and bleached residues are shown in Figure 4. All samples exhibited a first weight loss step at 25 and 125 °C corresponding to the loss of bonded water and the typical degradation steps of lignocellulosic residues, previously commented. The TGA curves of the bleached fractions showed the expected differences in the thermal behavior with respect to the non-bleached samples, related to the compositional changes that occurred in the bleaching step. The partial removal of hemicellulose during the bleaching cycles is reflected on the TGA curves, where the double peak in DTGA curves of polysaccharides became a single peak, mainly attributed to cellulose degradation, and the temperature of the maximum degradation rate increased from 280 to 300 °C in BR-110 and BR-130 samples. Nevertheless, no relevant changes in the cellulose purification degree can be deduced from the scarce increase in the weight loss step attributed to this polymer, remaining other compounds whose degradation overlapped with the cellulose degradation, as also observed in the analyzed composition. In sample BR-170, very few changes in the TGA curve were observed after the first bleaching cycle, coherently with the small composition changes reflected in Table 3. Therefore, the successive cycles reduced the bleaching mass yield but did not significantly promote cellulose purification but its degradation. In the other cases, the bleaching cycles promoted the removal of hemicellulose but also did not result in higher cellulose purity due to its partial degradation. The cellulose degradation products probably contributed to the increase in the final residual mass obtained for most of the bleached samples. Therefore, the oxidative process applied with hydrogen peroxide in an alkaline medium seems to partially degrade cellulose, generating other compounds and reducing the process yield.

Subcritical water extraction of BSG represents an innovative technique to valorize this waste. On one hand, phenolic-rich fractions are obtained with antioxidant and antimicrobial activities and high protein content. On the other hand, cellulose-rich solid residues are generated, which can be further purified by means of chlorin-free solvents using a most sustainable and environmentally friendly process. The selection of the temperature allows for modulating the protein and phenolic richness of the extracts. At 170 °C, the highest protein and phenolic contents were obtained. However, the cellulose purification reached its highest yield in the samples obtained at 150 °C. Therefore, the use of SWE allows the efficient fractionation of the BGS depending on the applied temperature.

## 3. Materials and Methods

### 3.1. Materials 

Petroleum ether (40–60 °C bp), phosphorous pentoxide (P_2_O_5_, 98.2%), sodium hydroxide (NaOH), glucose, and arabinose were purchased from Sigma-Aldrich (St. Louis, MO, USA). D (+)-Xylose was supplied by Merck KGaA (Darmstadt, Germany). Ethanol (98%), hydrogen peroxide (H_2_O_2_, 30%), sulphuric acid (H_2_SO_4_, 98%), and sodium carbonate (Na_2_CO_3_, 99.5%) were obtained from Panreac Quimica S.L.U. (Castellar del Vallés, Barcelona, Spain).

### 3.2. Residue Preparation 

Beer bagasse, supplied by a brewery factory located in Valencia, was dried at 60 °C ± 2 °C in a forced-air oven (J.P. Selecta, Barcelona, Spain) until constant weight. After that, it was milled using a Thermomix (Model TM6 Vorwerk, Wuppertal, Germany) and sieved to obtain particles under 0.71 mm and cold stored.

The defatting process was performed under reflux with petroleum ether for 8 h at 60 °C, with stirring using a 1:4 ratio of dry sample to solvent. Then, it was allowed to settle, decanted, and filtered with a 125-mm paper filter, washing with pure solvent, to separate the defatted residue that was left to solvent evaporation at room temperature in an extractor hood for 16 h, when constant weight was reached. The liquid phase was dried by adding anhydrous sodium sulfate and then left for 48 h and filtered under vacuum. The oil was recovered by evaporating petroleum ether in the vacuum rotary evaporator (Rotary Evaporators, Heidolph Instruments GmbH & Co. KG, Walpersdorfer, Germany). 

### 3.3. Subcritical Water Extraction

Subcritical extraction (SWE) of defatted bagasse was carried out with a ratio solids-water of 1:8 using a pressure reactor (Model 1-TAP-CE, 5 L capacity, Amar Equipment PVT. LTD, Mumbai, India). The temperature-pression conditions used were 110 °C-1 bar, 130 °C-2 bar, 150 °C-4.5 bar, and 170 °C-8 bar, applying 50 rpm in all cases, for 30 min. After each extraction step, the defatted sample dispersions were filtered through a filter with a pore size less than 0.5 mm (Filterlab, Barcelona, Spain). Thus, two fractions were obtained from each SWE process: one insoluble residue (R) and the soluble extracts (E). The extracts, named E-110, E-130, E-150, and E-170, were lyophilized at −60 °C and 0.8 mbar and stored in desiccators (P_2_O_5_, 0% relative humidity) at 4 °C. The respective mass yields of extracted solids and solid residues were determined with respect to the initial defatted bagasse. To determine the extract yield, three aliquot samples of the liquid extracts were dried at 105 °C until a constant weight was determined to determine the water:solid ratio, and the total solids extracted was calculated by multiplying by the total water mass in the reactor. To determine the yield in the extraction residues (R-110, R-130, R-150, and R-170), these were washed with distilled water, filtered, and dried at 40 °C for 48 h to determine their weight yield; then these were stored at 4 °C until further use.

### 3.4. Bleaching Process

The insoluble fractions obtained from SWEs were bleached following the method described by [26], using hydrogen peroxide as a bleaching agent. 4% (wt) H_2_O_2_ solution was prepared while pH was fitted to 12 (using NaOH). The lignocellulosic residues were treated with this bleaching solution using a solvent-solid ratio of 30:1 at 40 °C for 1 h in four consecutive cycles, filtering and washing the sample with distilled water after each cycle.

After the four cycles, the cellulose fractions were filtered and washed with abundant deionized water to remove residues of the bleaching solution and then dried at 50 °C overnight. The bleached samples were labeled BR-110-1C to BR-110-4C, BR-130-1C to BR-130-4C, BR-150-1C to BR-150-4C, and BR-170-1C to BR-170-4C. The mass yield (%) and whiteness index (WI) were determined in all samples using Equations (1) and (2) at each bleaching cycle to control the sample development. The color coordinates L* (lightness), a* (red green), and b* (yellowish-blue) of each bleached fraction were obtained with a CM-3600d spectro-colorimeter (Minolta Co., Tokyo, Japan), using a D65 illuminant and 10^0^ observed.

(1) $$Yield=\frac{Weight\;of\;bleached\;cellulose}{Weight\;of\;material\;before\;bleaching}$$

(2) $$WI=100-\sqrt{{\left(100-L*\right)}^{2}+{a*}^{2}+{b*}^{2}}$$

### 3.5. Physico-Chemical Analysis of Beer Bagasse, Soluble, and Insoluble Fractions

The amount of protein in raw beer bagasse BB, extracts (E-110, E-130, E-150, and E-170), and extraction residues (R-110, R-130, R-150, and R-170) was measured using the Dumas combustion method (Leco, St. Joseph, MI, USA) by duplicate. A conversion factor of 4.74 was applied to calculate protein content from total nitrogen [50].

In the same way, all samples were subjected to thermogravimetric analysis (TGA). A TGA/SDTA 851e analyzer (Mettler Toledo, Schwarzenbach, Switzerland) working under nitrogen flow (20 mL/min) was used to obtain the weight loss vs. temperature curves (TGA) and the first derivatives (DTGA). Samples (3–5 mg) of a previously conditioned sample in P_2_O_5_ were placed in a 70-μL alumina crucible and heated from 25 to 900 °C at 10 K/min. Three replicates per sample were obtained.

#### 3.5.1. Analysis of Structural Components in the Insoluble Fraction 

Cellulose, hemicellulose, acid-insoluble lignin content of defatted beer bagasse (DB), insoluble fractions (R), and bleached samples (BR) were analyzed according to the method of the National Renewable Energy Laboratory (NREL/TP-510-42618—2008) [46]. The test consisted of a two-stage hydrolysis with 72% sulfuric acid, of which one results in a soluble fraction in which the sugar content (glucose, xylose, and arabinose) was measured by high-resolution liquid chromatography. (HPLC, Agilent Technologies, model 1120 Compact LC, Waldbronn, Germany) and a RezexTM RCM-Monosaccharide Ca^2+^ column (300 × 7.8 mm). On the other hand, the insoluble fraction was used to quantify the acid-insoluble lignin content by the thermogravimetric method. The cellulose content was obtained from the quantified glucose and hemicellulose from the sum of quantified xylose and arabinose.

Before hydrolysis, the raw material (DB) and the insoluble fractions (R) were subjected to the extractive determination using the standard NREL method (NREL/TP-510-42619—2008) [47]. This procedure was performed using a Soxhlet set-up, which consists of two stages: a first extraction with water for 6 h, followed by a second extraction with ethanol at 60 °C for 6 h.

The thermal stability analysis was carried out on all samples by triplicate using the TGA 1 Stare System analyzer (Mettler Toledo, Greifensee, Switzerland), previously conditioned in phosphorous pentoxide (P_2_O_5_) for two weeks. The analysis was conducted from 25 to 900 °C at 10 K/min with a nitrogen flow of 10 mL per minute. In addition, the ash content was analyzed using the UNE-EN 14775 standard.

#### 3.5.2. Total Phenolic Content, Antioxidant and Antibacterial Properties of Soluble Fraction

The total phenol content was determined using the Folin Ciocalteu method. Briefly, 0.5 mL of each extract was mixed with 6 mL of distilled water, and 0.5 mL of Folin reagent (2N) was added. After one minute, 1.5 mL of a 20% Na_2_CO_3_ solution and distilled water were added to a volume of 10 mL. After stirring, they were kept in the dark for 2 h. The absorbance at 725 nm was then measured in triplicate using a UV-Vis spectrophotometer (Evolution 201, Thermo Scientific, Waltham, MA, USA). Gallic acid was used as a standard, and the results were expressed as mg L^−1^ gallic acid equivalents (GAE) using a standard curve (r^2^ = 0.9991) of gallic acid (2–20 mg·L^−1^). The TPC value of milled raw material was also determined by previously extracting phenols with methanol at a solid to liquid ratio of 1:50 at room temperature and dark for 24 h under constant stirring.

The antiradical capacity of the extracts was determined using the 2,2-diphenyl-1-pikryl-hydrazyl (DPPH) free radical method [51]. For each extract, a solution of DPPH in methanol at a concentration of 6.22 × 10^−2^ mM was mixed with different extract concentrations until reaching a final volume of 4 mL. The resulting solutions were kept in the dark at room temperature for 12 h, after which the absorbance at 515 nm was measured. The initial and final concentrations of DPPH in the reaction medium were calculated from a calibration curve fitted by linear regression (R^2^ = 0.9992). The antiradical activity was evaluated by the EC_50_ parameter, which represents the amount of antioxidants necessary to reduce the initial concentration of DPPH by 50% when the stability of the reaction is reached. This value was expressed as mg of dried extract per mg of DPPH and also in mg defatted beer bagasse per mg of DPPH for comparison purposes. EC_50_ values were determined from graphs showing the percentage of [DPPH] remaining as a function of the amount of solid extract relative to the amount of DPPH, using Equation (3):

(3) $$\%{\left[DPPH\right]}_{remaining}=\frac{{\left[DPPH\right]}_{t}}{{\left[DPPH\right]}_{t=0}}\times 100$$

Regarding the antimicrobial capacity of the extracts, the minimum inhibitory concentration (MIC) of the different extracts was determined for two bacteria: the Gram-positive bacterium *Listeria innocua* and the Gram-negative *Escherichia coli*. This analysis followed the method outlined by [26], using standard 96-well microtiter plates with a total volume of 200 µL. For both bacterial strains stored at −20 °C, a stock solution was prepared by transferring bacterial amounts using an inoculation loop to a volume of 10 mL of TSB and incubated at 37 °C for 24 h. Subsequently, 10 µL of the stock solution was taken and transferred to a tube containing 10 mL of TSB to prepare the corresponding work solution with a concentration of 10^5^ CFU·mL^−1^. This concentration was confirmed through serial dilution and counting. For each bacterium, 100 µL of the bacterial solution with an initial concentration of 10^5^ CFU.mL^−1^ was added to each well. Thereafter, different volumes of each extract solution, with 200 mg.mL^−1^, were added in each well, while the final volume was adjusted to 200 µL with TSB to obtain different extract dilutions. The plates were then incubated at 37 °C for 24 h. Afterward, 100 µL from each well was transferred to TSA plates and incubated at 37 °C for 24 h for final counting. The MIC for each extract was determined as the lowest extract concentration at which no bacterial growth was observed on the TSA plate. This analysis was performed in duplicate.

### 3.6. Statistical Analysis

The Statgraphics Centurion XVII-64 program version 19 (Manugistics Corp., Rockville, MD, USA) was used to perform statistical analyses using an analysis of variance (ANOVA) with a confidence level of 95%. The Fisher test was applied to detect possible differences in treatment responses, using a critical value of 5% to determine significance.

## 4. Conclusions

Despite being rich in polysaccharides, proteins, and phenolic compounds, BSG is still underutilized in the food, materials, or pharmaceutical sectors. New sustainable approaches, such as the use of subcritical water extraction, could be a possible technology to fractionate this waste, obtaining bioactive agents, proteins, and cellulose fractions from the beer bagasse while contributing to the circular economy.

The use of subcritical water treatment of defatted beer bagasse allowed to obtain bioactive aqueous extracts (7–41% mass yield of the defatted BSG) with radical scavenging capacity and antimicrobial activities. The highest extraction temperature (170 °C) gave rise to the highest extract yield while providing the extracts with greater polyphenol content and antibacterial effect but with lower DPPH radical scavenging capacity. In contrast, the extraction at 150 °C was optimal for producing extracts (35% mass yield) with the greatest radical scavenging capacity. Likewise, the extract obtained at 170 °C was the richest in protein, which could be separated by precipitation from the liquid extract.

Beer bagasse can be considered a relatively poor source of cellulose in comparison with other agro-industrial residues. Considering the yield (g cellulose fraction/100 g DB) of the different process steps to purify cellulose (19, 17, 14, and 13% for samples treated at 110, 130, 150, and 170 °C) and the similar degree of cellulose purity obtained after the 4 bleaching cycles (50, 60, 70, and 50%, respectively, for samples treated at 110, 130, 150, and 170 °C), the best treatment to obtain cellulose would be the extraction at 150 °C, followed by two bleaching cycles with hydrogen peroxide. These conditions allowed for the removal of most of the hemicellulose and led to a cellulose purity degree without significant differences with respect to that obtained in the successive cycles.

Thus, the subcritical water extraction method highlights the potential of simple processes as a technological option to convert underutilized side streams like beer bagasse into added-value, potential ingredients for innovative food and pharmaceutical applications.

## Figures and Tables

**Table 1:** Chemical composition of defatted beer bagasse (DB) and insoluble fractions after SWE at different temperatures.

Sample	Extractive (%)	Protein (%)	Ash (%)	Lignin * (%)	Cellulose (%)	Hemicellulose (%)
DB	13.1 ± 0.5 ^a^	22 ± 2 ^a^	3.71 ± 0.01 ^c^	9.5 ± 1.6 ^a^^b^	17 ± 2 ^a^	17.9 ± 0.6 ^d^
R-110	20.9 ± 1.5 ^b^	28 ± 2 ^b^	3.12 ± 0.11 ^b^	9.1 ± 0.4 ^a^	16 ± 2 ^a^	15 ± 2 ^c^
R-130	21.79 ± 0.05 ^b^	26.2 ± 0.2 ^ab^	2.79 ± 0.04 ^b^	11.56 ± 0.08 ^b^	20 ± 2 ^a^	14.9± 1.2 ^c^
R-150	34.5 ± 0.9 ^d^	26 ± 2 ^b^	2.27 ± 0.11 ^a^	14.6 ± 0.5 ^c^	21 ± 2 ^a^	7.8 ± 1.2 ^b^
R-170	28 ± 3 ^c^	35.7 ± 0.2 ^a^^b^	3.1 ± 0.2 ^b^	19.6 ± 0.3 ^d^	30 ± 3 ^b^	2.01 ± 0.08 ^a^

^a–d^ different superscript letters in the same column indicate significant differences (*p* < 0.05); * acid insoluble lignin.

**Table 2:** Total phenolic content (TPC), efficient concentration (EC_50_), protein content, and ashes of the aqueous extracts (E) (dry basis) obtained from the SWE process at different temperatures. (Mean values ± standard deviation).

	E-110	E-130	E-150	E-170
Protein (g/100 g extract)	15.1 ± 0.1 ^a^	16.6 ± 0.2 ^a^	22.4 ± 1.2 ^b^	28.7 ± 0.6 ^c^
Ashes (g/100 g extract)	1.54 ± 0.06 ^c^	1.5 ± 0.1 ^c^	1.20 ± 0.04 ^b^	0.46 ± 0.01 ^a^
TPC_1_ (mg GAE/g extract)	16.8 ± 0.1 ^a^	22 ± 2 ^a^	17.91 ± 0.07 ^b^	59.1 ± 0.2 ^c^
TPC_2_ (mg GAE/g DB)	1.27 ± 0.08 ^a^	3.2 ± 0.3 ^ab^	6.34 ± 0.02 ^ab^	24.18 ± 0.08 ^b^
EC_50_ (mg extract/mg DPPH)	13 ± 3 ^a^	18 ± 2 ^a^	39 ± 4 ^b^	59 ± 0.5 ^c^
MIC (mg/mL) against *L. innocua*	264	198	168	80
MIC (mg/mL) against *E. coli*	234	204	162	140

^a–c^ different superscripts in the same row indicate significant differences among extracts (*p* < 0.05).

**Table 3:** Chemical composition (wt%) of the insoluble fractions subjected to the four bleaching cycles with 4% hydrogen peroxide.

Sample	Ashes (%)	Lignin (%)	Protein (%)	Cellulose (%)	Hemicellulose (%)
BR-110-1C	6.96 ± 0.09 ^a,1^	14.69 ± 0.03 ^a,1^	11.3 ± 0.5 ^a,3^	52 ± 2 ^a,1^	41 ± 5 ^a,1^
BR-110-2C	5.9 ± 0.3 ^b,1^	15.9 ± 0.8 ^a,2^	8.2 ± 0.3 ^b,2^	44 ± 2 ^a,1^	33 ± 4 ^b,1^
BR-110-3C	6.20 ± 0.08 ^b,1^	13.88 ± 1.09 ^a,2^	5,1 ± 0.2 ^c,2^	57 ± 5 ^a,1^	30 ± 3 ^c,1^
BR-110-4C	6.2 ± 0.3 ^b,1^	11.5 ± 0.9 ^b,1^	1.9 ± 0.3 ^d,2^	53 ± 4 ^a,1^	26 ± 3 ^d,1^
BR-130-1C	6.5 ± 0.3 ^b,1^	16.2 ± 0.5 ^ab,2^	21 ± 0.6 ^a,1^	62 ± 3 ^a,1^	28 ± 3 ^a,2^
BR-130-2C	7.07 ± 0.07 ^b,2^	17.3 ± 0.4 ^bc,1^	11.3 ± 0.4 ^b,1^	65 ± 4 ^a,2^	31 ± 6 ^a,1^
BR-130-3C	6.5 ± 0.3 ^b,1^	17.97 ± 0.04 ^c,3^	7.7 ± 0.2 ^c,1^	67± 3 ^a,1^	22 ± 3 ^ab,2^
BR-130-4C	5.8 ± 0.2 ^a,1^	15.6 ± 0.8 ^a,2^	2.5 ± 0.1 ^d,1^	62 ± 5 ^a,12^	15 ± 2 ^b,2^
BR-150-1C	5.07 ± 0.41 ^a,2^	20.88 ± 0.06 ^a,3^	16.2 ± 0.9 ^a,2^	58 ± 6 ^a,1^	15 ± 2 ^a,3^
BR-150-2C	7.4 ± 0.5 ^a,2^	15.35 ± 0.11 ^b,23^	11.6 ± 0.4 ^b,1^	64 ± 5 ^a,2^	14 ± 2 ^a,2^
BR-150-3C	5.5 ± 1.4 ^a,1^	15 ± 2 ^b,2^	7.3 ± 0.2 ^c,1^	62 ± 5 ^a,1^	-
BR-150-4C	4.9 ± 0.7 ^a,1^	14.77 ± 1.02 ^b,2^	2.9 ± 0.7 ^d,1^	71 ± 6 ^a,2^	-
BR-170-1C	5.5 ± 0.4 ^a,2^	21.9 ± 0.3 ^a,4^	6.7 ± 0.2 ^a,4^	60 ± 3 ^a,1^	-
BR-170-2C	5.7 ± 0.3 ^a,1^	14.4 ± 0.2 ^b,3^	4.8 ± 0.3 ^b,3^	42 ± 2 ^c,1^	-
BR-170-3C	5.7 ± 0.4 ^a,1^	11.5 ± 0.2 ^c,1^	2.9 ± 0.3 ^c,3^	44 ± 2 ^c,2^	-
BR-170-4C	6.2 ± 0.2 ^a,1^	10.2 ± 0.2 ^d,1^	1.2 ± 0.2 ^d,3^	53 ± 4 ^b,1^	-

^a–d^ different letters indicate significant differences (*p* < 0.05) between samples at the same extraction temperature; ^1–4^: different numbers indicate significant differences (*p* < 0.05) between samples at the same bleaching cycle.

## Data Availability

Data is contained within the article.

## Supplementary Materials

The following supporting information can be downloaded at: https://www.mdpi.com/article/10.3390/molecules29204897/s1, Table S1: Chemical composition of defatted beer bagasse (DB) and insoluble fractions after SWE at different temperatures (g/100 g initial defatted bagasse); Table S2: Chemical composition (g/100 g initial defatted bagasse) of the insoluble fractions subjected to the four bleaching cycles with 4% hydrogen peroxide.
