# Supplementary material for: Applying Subcritical Water Extraction to Obtain Bioactive Compounds and Cellulose Fibers from Brewer Spent Grains
Source: Molecules. 2024 Oct 16;29(20):4897. doi: 10.3390/molecules29204897 (PMC11510227; doi:10.3390/molecules29204897)
Supplement: Supplementary file 1 [file molecules-29-04897-s001.zip › molecules-3250667-supplementary.pdf]

Table s1. Chemical composition of defatted beer bagasse (DB) and insoluble fractions after SWE at different temperatures (g /100 g initial defatted bagasse)

Sample	Protein (%)	Ash (%)	Lignin (%)	Cellulose (%)	Hemi-cellulose (%)
DB	22.0 ± 2[a]	3.71 ± 0.01[a]	9.5 ± 1.6[a]	17 ± 2[a]	17.9 ± 0.6[a]
R-110	24.8 ± 1.8[a]	2.8 ± 0.1[b]	8.3 ± 0.4[a]	17 ± 2[ab]	13.3 ± 1.5[b]
R-130	21.4 ± 0.2[b]	2.3 ± 0.03[c]	9.5 ± 0.1[a]	17 ± 2[ab]	12.4 ± 1[b]
R-150	16.6 ± 1.3[c]	1.5 ± 0.07[d]	9.4 ± 0.3[a]	14 ± 1.4[c]	5.0 ± 0.7[c]
R-170	13.2 ± 0.1[d]	1.6 ± 0.1[e]	10.0 ± 0.2[b]	16 ± 1.5[ab]	1.0 ± 0.04[d]

[a,b,c,d,e] different letters in the same column indicate significant differences among samples at 95%

Table s2. Chemical composition (g/100 g initial defatted bagasse) of the insoluble fractions subjected to the four bleaching cycles with 4% hydrogen peroxide.

Sample	Ashes (%)	Lignin (%)	Protein (%)	Cellulose (%)	Hemi-cellulose (%)
BR-110-1C	3.0 ± 0.04[a]	6.4 ± 0.01[a]	3.7 ± 0.22[a]	22.8 ± 0.9[a]	17.8 ± 2.2[a]
BR-110-2C	1.5 ± 0.08[b]	4.0 ± 0.2[b]	1.6 ± 0.08[b]	11.3 ± 0.5[b]	8.5 ± 1[b]
BR-110-3C	1.4 ± 0.02[b]	3.2 ± 0.25[c]	0.9 ± 0.05[c]	13.3 ± 1.2[b]	7.0 ± 0.7[b]
BR-110-4C	1.2 ± 0.06[b]	2.2 ± 0.17[d]	0.3 ± 0.06[d]	10.0 ± 0.8[b]	4.9 ± 0.6[b]
BR-130-1C	2.7 ± 0.12[a]	6.7 ± 0.21[a]	6.6 ± 0.6[a]	13.1 ± 1.2[a]	11.6 ± 1.2[a]
BR-130-2C	2.1 ± 0.02[b]	5.1 ± 0.12[b]	2.5 ± 0.4[b]	17.0 ± 1.2[a]	9.1 ± 1.8[a]
BR-130-3C	1.5 ± 0.07[c]	4.0 ± 0.01[c]	1.3 ± 0.2[c]	14.9 ± 0.7[a]	5.0 ± 0.7[a]
BR-130-4C	1.0 ± 0.03[d]	2.7 ± 0.14[d]	0.3 ± 0.1[d]	13.4 ± 0.9[a]	2.5 ± 0.3[a]
BR-150-1C	1.5 ± 0.12[a]	6.0 ± 0.02[a]	3.5 ± 0.9[a]	16.6 ± 1.7[a]	4.3 ± 0.6[a]
BR-150-2C	1.7 ± 0.11[a]	3.5 ± 0.02[b]	2.0 ± 0.4[b]	14.5 ± 1.1[b]	3.2 ± 0.5[c]
BR-150-3C	0.9 ± 0.24[b]	2.6 ± 0.34[c]	1.0 ± 0.2[b]	10.7 ± 0.9[b]	0.0[c]
BR-150-4C	0.7 ± 0.1[b]	2.1 ± 0.15[c]	0.3 ± 0.7[c]	10.3 ± 0.7[b]	0.0[c]
BR-170-1C	1.1 ± 0.08[a]	9.1 ± 0.06[a]	1.0 ± 0.2[a]	11.7 ± 1.2[a]	0.0
BR-170-2C	0.8 ± 0.04[b]	4.2 ± 0.03[b]	0.5 ± 0.3[b]	6.2 ± 0.4[b]	0.0
BR-170-3C	0.8 ± 0.06[b]	2.6 ± 0.03[c]	0.3 ± 0.3[b]	6.1 ± 0.3[b]	0.0
BR-170-4C	0.8 ± 0.03[b]	1.7 ± 0.03[d]	0.1 ± 0.2[b]	6.8 ± 0.3[b]	0.0

[a,b,c,d] different letters at the same temperature indicate significant differences among cycles at 95%
